# Risk of long-term renal disease in women with a history of preterm delivery: a population-based cohort study

**DOI:** 10.1186/s12916-020-01534-9

**Published:** 2020-04-01

**Authors:** Peter M. Barrett, Fergus P. McCarthy, Marie Evans, Marius Kublickas, Ivan J. Perry, Peter Stenvinkel, Karolina Kublickiene, Ali S. Khashan

**Affiliations:** 1 School of Public Health, Western Gateway Building, University College Cork, Cork, Ireland; 2 Irish Centre for Maternal and Child Health Research, Cork University Maternity Hospital, University College Cork, Cork, Ireland; 3 Department of Obstetrics & Gynaecology, Cork University Maternity Hospital, Cork, Ireland; 4 Division of Renal Medicine, Department of Clinical Intervention, Science and Technology (CLINTEC), Karolinska Institutet, Karolinska University Hospital, Stockholm, Sweden; 5 Department of Obstetrics & Gynaecology, Karolinska University Hospital, Stockholm, Sweden

**Keywords:** Preterm delivery, Chronic kidney disease, End-stage kidney disease, Pregnancy, Preeclampsia, Epidemiology

## Abstract

**Background:**

Preterm delivery is an independent risk factor for maternal cardiovascular disease. Little is known about the association between preterm delivery and maternal renal function. This study aimed to examine whether women who experience preterm delivery are at increased risk of subsequent chronic kidney disease (CKD) and end-stage kidney disease (ESKD).

**Methods:**

Using data from the Swedish Medical Birth Register, singleton live births from 1973 to 2012 were identified and linked to data from the Swedish Renal Register and National Patient Register (up to 2013). Gestational age at delivery was the main exposure and treated as a time-dependent variable. Primary outcomes were maternal CKD or ESKD. Cox proportional hazard regression models were used for analysis.

**Results:**

The dataset included 1,943,716 women who had 3,760,429 singleton live births. The median follow-up was 20.6 (interquartile range 9.9–30.0) years. Overall, 162,918 women (8.4%) delivered at least 1 preterm infant (< 37 weeks). Women who had any preterm delivery (< 37 weeks) were at increased risk of CKD (adjusted hazard ratio (aHR) 1.39, 95% CI 1.32–1.45) and ESKD (aHR 2.22, 95% CI 1.90–2.58) compared with women who only delivered at term (≥ 37 weeks). Women who delivered an extremely preterm infant (< 28 weeks) were at increased risk of CKD (aHR 1.84, 95% CI 1.52–2.22) and ESKD (aHR 3.61, 95% CI 2.03–6.39). The highest risk of CKD and ESKD was in women who experienced preterm delivery + preeclampsia (vs. non-preeclamptic term deliveries, for CKD, aHR 2.81, 95% CI 2.46–3.20; for ESKD, aHR 6.70, 95% CI 4.70–9.56). However, spontaneous preterm delivery was also associated with increased risk of CKD (aHR 1.32, 95% CI 1.25–1.39) and ESKD (aHR 1.99, 95% CI 1.67–2.38) independent of preeclampsia or small for gestational age (SGA).

**Conclusions:**

Women with history of preterm delivery are at increased risk of CKD and ESKD. The risk is higher among women who had very preterm or extremely preterm deliveries, or whose preterm delivery was medically indicated. Women who experience spontaneous preterm delivery are at increased risk of long-term renal disease independent of preeclampsia or SGA. Preterm delivery may act as a risk marker for adverse maternal renal outcomes.

## Background

Chronic kidney disease (CKD) is a major cause of morbidity globally, with an estimated prevalence of 7–12% for stage 3 CKD or greater among women [1–3]. End-stage kidney disease (ESKD), although relatively rare, has a disproportionately high healthcare burden. Preterm delivery, before 37 weeks’ gestation, is recognised as a risk factor for maternal cardiovascular disease (CVD), independent of socio-demographic factors (e.g. age, ethnicity, education), obstetric history (e.g. preeclampsia, parity), and cardiometabolic factors (e.g. chronic hypertension, diabetes, body mass index (BMI)) [4–6]. Although cardiovascular guidelines suggest that pregnancy-related factors, including preterm delivery, should be considered markers of future CVD risk [7, 8], little is known about the association between preterm delivery and subsequent renal disease. This information could potentially provide opportunities for risk stratification and secondary prevention of CKD in women.

Existing research on preterm delivery and the risk of maternal CKD has been limited to groups with an increased baseline risk of renal disease [9, 10], or has failed to adjust for important confounders such as maternal smoking [11–13] and obesity [12, 13]. Cardiovascular risk is higher among women who experience very or extremely preterm deliveries (before 32 weeks’ gestation) [4], but it is uncertain whether a similar pattern exists for future risk of CKD. It is plausible that women with earlier preterm deliveries experience greater risk of CKD or ESKD relative to women who deliver closer to full term.

Most preterm deliveries occur spontaneously, but about 30% are iatrogenic and performed for obstetric reasons, typically maternal preeclampsia or intrauterine growth restriction (IUGR) [14]. Preeclampsia has been identified as a strong risk factor for ESKD [11, 13, 15], and women who experience IUGR or a small for gestational age (SGA) delivery may also be at increased risk of CKD [11, 16]. Thus, the risk of maternal CKD/ESKD may differ depending on whether women experience spontaneous or iatrogenic preterm deliveries.

We aimed to examine whether women who experience preterm delivery are at increased risk of CKD and ESKD, and whether this association differs by medical indication (i.e. spontaneous or iatrogenic preterm) or across categories of gestational age.

## Methods

### Study population

Data from the Swedish Medical Birth Register (MBR) (established 1973) were used to identify all women who had singleton live births between January 1, 1973, and December 31, 2012. Data from the Swedish National Patient Register (NPR) (established 1964) and Swedish Renal Register (SRR) (established 1991) were linked with the MBR using anonymised unique identification numbers. Data from the NPR and SRR were used to identify women who developed CKD or ESKD during follow-up, until December 31, 2013 (study end date). Data from the Swedish Death Register and Migration Register were also available until December 31, 2013, and used for censoring (Additional file 1. Supplementary Figure S1).

In order to reduce potential confounding from comorbid diseases, we identified women with pre-pregnancy CKD, ESKD, CVD, hypertension, diabetes (type 1 or 2), or systemic lupus erythematosus (SLE) from the MBR, and they were excluded from the study. We also excluded all women in the NPR who were admitted to hospital with these diagnoses before their index pregnancy. Three iterations of ICD coding were used to identify pre-existing diseases in the NPR: ICD-8 coding before 1986, ICD-9 coding from 1987 to 1996, and ICD-10 coding from 1997 to 2013. New hospital admissions and outpatient reviews for preeclampsia and gestational diabetes were also identified in the NPR using ICD codes, and used to supplement information in the MBR. The ICD codes are summarised in Additional file 2 (Supplementary Table S1).

We further excluded multiple pregnancies, stillbirths, and pregnancies with implausible dates of delivery from all analyses (Additional file 3. Supplementary Figure S2).

### Preterm delivery

Maternal history of preterm delivery was the main exposure of interest. Gestational age at delivery was estimated based on second trimester ultrasound (around 17th week of pregnancy) where available, or the time from last menstrual period (LMP) to birth based on maternal report at first antenatal visit. LMP was used to estimate gestational age until 1982, but ultrasonography data became increasingly available in the MBR thereafter.

Five separate exposure variables were used to investigate the effects of gestational age, including preterm and early term delivery, on maternal CKD/ESKD risk. Each of these used different classifications of preterm delivery. For all models, time-dependent variables were used so that a woman could contribute pregnancies and person-time to both unexposed and exposed groups during follow-up. Women could contribute unexposed time if they never had a preterm delivery, or until their first preterm delivery occurred. For example, if a woman delivered at term (≥ 37 weeks) in the first pregnancy, and then had a preterm delivery (< 37 weeks) in the second pregnancy, she was considered unexposed between deliveries 1 and 2, and exposed from delivery 2 onwards irrespective of subsequent pregnancy outcomes.

For the main analysis (exposure 1), women were categorised according to any previous history of preterm delivery (ever, < 37 weeks). If women had more than one pregnancy, they were considered exposed from the date of their first preterm delivery onwards. For exposure 2, established definitions of term (≥ 37 weeks, reference group) vs. moderate (32–36 + 6 weeks)/very (28–31 + 6 weeks)/extremely (< 28 weeks) preterm were used. Maternal exposure status was always based on the earliest gestation of any previous delivery. For example, if a woman had three deliveries during follow-up, at 35 weeks, 27 weeks, and 39 weeks, respectively, she contributed exposed time to the moderate preterm category between deliveries 1 and 2, and contributed exposed time to the extremely preterm category thereafter (irrespective of her third delivery at term).

Next, for exposure 3, additional categories of early term deliveries (37–38 + 6 weeks) and post-term deliveries (≥ 42 weeks) were used together with the established categories of moderate/very/extremely preterm. The reference group for exposure 3 was those who delivered at “full term” (i.e. 39–41 + 6 weeks). Early term deliveries (37–38 + 6 weeks) were examined separately since they are associated with increased risk of adverse perinatal outcomes [17, 18], and they share common risk factors with preterm deliveries [19].

For exposure 4, preterm deliveries (< 37 weeks) were stratified by spontaneous or iatrogenic preterm, where those deliveries complicated by preeclampsia and/or SGA were assumed to be iatrogenic preterm. A series of dummy variables were included to represent non-overlapping scenarios: (i) preterm delivery alone (i.e. assumed spontaneous preterm), (ii) preterm delivery + preeclampsia, (iii) preterm delivery + SGA, and (iv) preterm delivery + preeclampsia + SGA (co-occurring). The definition of SGA in the MBR was a birth weight of 2 standard deviations (SD) below the sex-specific and gestational age distributions, according to Swedish weight-based growth standards [20].

Finally, for exposure 5, the analysis was restricted to all women in the dataset who only had two live births during the study period to examine differences in risk between none/one/two preterm deliveries (< 37 weeks).

### Outcome variables

There were two main outcomes: maternal CKD and maternal ESKD. Outcome data were defined by a recorded diagnosis of CKD or ESKD in the SRR or based on a primary or secondary diagnosis of CKD or ESKD in the NPR (using ICD codes). The earliest date at which a woman appeared in either the SRR or the NPR was assumed to be her date of diagnosis for CKD/ESKD. Women who had renal disease due to an identifiable congenital or genetic cause were excluded (Additional file 2. Supplementary Table S1).

### Covariates

The following covariates were adjusted for: maternal age, year of delivery, country of origin, education level, body mass index (BMI), smoking during pregnancy, gestational diabetes, preeclampsia, parity, and inter-pregnancy interval. Education level was available from the Swedish Education Register and was based on the mother’s highest level of educational achievement (proxy variable for socio-economic status). Smoking status was based on any reported smoking during pregnancy, either at first antenatal visit or at 30–32 weeks’ gestation. Maternal BMI was calculated based on weight and length (kg/m^2^) as recorded at first antenatal visit. There were large amounts of missing data for smoking status and BMI, and these variables were only available from 1982 onwards. Missing indicator variables were created to control for this. A sensitivity analysis was also undertaken where the dataset was restricted to births between 1982 and 2012 to check for comparability with the main results.

Maternal exposure to gestational diabetes was treated as a time-dependent covariate, where women were considered exposed from their date of first delivery with gestational diabetes. Preeclampsia was also considered a time-dependent covariate and was adjusted for in all models except when exposure 4 was used, since preterm preeclamptic deliveries were already considered as a separate group. Inter-pregnancy interval was defined as the date between a woman’s last live birth and her estimated date of conception for the next live birth (estimated by subtracting gestational age in days from the date of next live birth).

### Statistical analysis

Data were set up for survival analysis, where entry date in the study was the date of each woman’s first live birth. The association between history of preterm delivery and risk of maternal CKD was estimated using the Kaplan-Meier method. Differences in survival curves were estimated using logrank tests. We used multivariable Cox proportional hazard regression models to estimate minimally adjusted and fully adjusted hazard ratios (aHRs) and 95% confidence intervals (CI) for the associations between preterm delivery and maternal renal disease. We followed women from date of entry until date of CKD/ESKD diagnosis, date of death, date of emigration, or study end date (31 December 2013), whichever came first. Log cumulative hazard plots were used to check the adequacy of each Cox regression model, and year of delivery was included in all minimally adjusted models to ensure the assumption of proportional hazards was met.

Two-sided *P* values were used, and *P* < 0.05 denoted statistical significance. All analyses were performed using Stata version 15 (StataCorp LLC).

## Results

The study cohort consisted of 1,943,716 unique women who had 3,760,429 singleton pregnancies, followed up for a total of 42,341,527 person-years. The median follow-up was 20.6 years (interquartile range 9.9–30.0), and the maximum follow-up was 41.0 years. There were 162,918 women (8.4%) who experienced preterm delivery (< 37 weeks) at some point (Table 1). From 1973 to 2013, 18,001 women (0.9%) developed CKD, and 1268 (0.07%) developed ESKD.

**Table 1:** Maternal characteristics and pregnancy outcomes among women delivering between 1973 and 2012 in Sweden, stratified by exposure to preterm delivery before 37 weeks (*n* = 1,943,716)

	No preterm delivery, *n* (%) *N* = 1,780,798 (91.6)	Preterm delivery, *n* (%) *N* = 162,918 (8.4)
Age in years		
< 20	97,007 (5.5)	14,384 (8.8)
20–29	1,151,931 (64.7)	104,337 (64.0)
30–39	507,226 (28.5)	41,921 (25.7)
≥ 40	24,634 (1.4)	2276 (1.4)
Native country		
Sweden	1,509,135 (84.7)	138,441 (85.0)
Elsewhere	271,663 (15.3)	24,477 (15.0)
Education level		
Less than upper secondary	233,341 (13.1)	24,540 (15.1)
Upper secondary	800,419 (45.0)	77,826 (47.8)
Third level	709,299 (39.8)	57,702 (35.4)
Missing	37,739 (2.1)	2850 (1.8)
Body mass index in early pregnancy (kg/m^2^)		
Underweight, < 18.5	41,129 (2.3)	4830 (3.0)
Normal, 18.5–24.9	655,619 (36.8)	55,415 (34.0)
Overweight, 25–29.9	178,593 (10.0)	15,447 (9.5)
Obese, ≥ 30	64,011 (3.6)	6445 (4.0)
Missing	841,446 (47.3)	80,781 (49.6)
Maternal smoking		
No	950,672 (53.4)	83,138 (51.0)
Yes	187,197 (10.5)	21,404 (13.1)
Missing	642,929 (36.1)	58,376 (35.8)
Gestational diabetes (ever)		
No	1,764,968 (99.1)	160,133 (98.3)
Yes	15,830 (0.9)	2785 (1.7)
Preeclampsia (ever)		
No	1,709,392 (96.0)	141,702 (87.0)
Yes	71,406 (4.0)	21,216 (13.0)
Small for gestational age (SGA) (ever)		
No	1,700,028 (95.6)	140,572 (86.5)
Yes	77,671 (4.4)	22,008 (13.5)
Decade of first birth		
1973–1979	478,412 (26.9)	39,063 (24.0)
1980–1989	385,271 (21.6)	42,506 (26.1)
1990–1999	386,441 (21.7)	38,576 (23.7)
2000–2012	530,674 (29.8)	42,773 (26.3)
Parity		
1	610,494 (34.3)	38,564 (23.7)
2	793,672 (44.6)	70,936 (43.5)
3	291,217 (16.4)	35,819 (22.0)
4	64,811 (3.6)	11,970 (7.4)
5 or more	20,604 (1.2)	5629 (3.5)

### History of any preterm or early term delivery

Tables 2 and 3 summarise the results of the main analyses for CKD and ESKD, respectively. Women who had at least one preterm delivery (< 37 weeks) (exposure 1) were at significantly increased risk of long-term CKD (aHR 1.39, 95% CI 1.32–1.45) and ESKD (aHR 2.22, 95% CI 1.90–2.58).

**Table 2:** Hazard ratios for maternal chronic kidney disease by history of preterm delivery, among women delivering between 1973 and 2012 in Sweden (*n* = 1,943,716)

	Chronic kidney disease (*N* = 18,001)		
	*n*	Minimally adjusted	Fully adjusted
		HR (95% CI)	HR (95% CI)
Exposure 1			
Term delivery (≥ 37 weeks)	15,914	1.0	1.0
Preterm delivery (< 37 weeks)	2087	1.47 (1.40–1.53)	1.39 (1.32–1.45)
Exposure 2			
Term delivery (≥ 37 weeks)	15,914	1.0	1.0
Moderate preterm delivery (32–36 + 6 weeks)	1733	1.41 (1.34–1.48)	1.35 (1.28–1.41)
Very preterm delivery (28–31 + 6 weeks)	244	1.77 (1.56–2.01)	1.55 (1.36–1.76)
Extremely preterm delivery (< 28 weeks)	110	2.05 (1.70–2.48)	1.84 (1.52–2.22)
Exposure 3			
Full term delivery (39–41 + 6 weeks)	9134	1.0	1.0
Post-term delivery (≥ 42 weeks)	2237	0.94 (0.90–0.98)	1.04 (0.99–1.09)
Early term delivery (37–38 + 6 weeks)	4543	1.13 (1.09–1.17)	1.19 (1.15–1.24)
Moderate preterm delivery (32–36 + 6 weeks)	1733	1.44 (1.37–1.52)	1.43 (1.36–1.51)
Very preterm delivery (28–31 + 6 weeks)	244	1.81 (1.60–2.06)	1.65 (1.45–1.87)
Extremely preterm delivery (< 28 weeks)	110	2.11 (1.75–2.54)	1.96 (1.63–2.37)
Exposure 4			
Term delivery (≥ 37 weeks)	15,914	1.0	1.0
Spontaneous preterm delivery (< 37 weeks)	1571	1.32 (1.25–1.39)	1.32 (1.25–1.39)
Iatrogenic preterm delivery—preeclampsia (< 37 weeks)	230	2.82 (2.48–3.21)	2.81 (2.46–3.20)
Iatrogenic preterm delivery—SGA (< 37 weeks)	182	1.74 (1.50–2.00)	1.66 (1.44–1.93)
Iatrogenic preterm delivery—preeclampsia and SGA (< 37 weeks)	104	2.11 (1.74–2.56)	2.10 (1.73–2.55)
Exposure 5			
Two term deliveries (≥ 37 weeks)	6013	1.0	1.0
One preterm (< 37 weeks) and one term delivery (≥ 37 weeks)	723	1.51 (1.40–1.63)	1.34 (1.24–1.45)
Two preterm deliveries (< 37 weeks)	93	1.67 (1.36–2.05)	1.46 (1.19–1.79)

Hazard ratios represent separate Cox regression models for associations between preterm delivery and maternal chronic kidney disease. Each exposure variable (1–5) represents different categories used to define gestational age at delivery. For all categories, preterm delivery was a time-dependent variable, where exposure status was based on the earliest gestation of any previous delivery. Minimally adjusted models controlled for year of delivery. Fully adjusted models controlled for year of delivery, maternal age, country of origin, education level, parity, inter-pregnancy interval, maternal BMI, smoking in pregnancy, exposure to gestational diabetes (time-dependent), and preeclampsia (time-dependent). In the analyses involving exposure 4 (spontaneous vs. iatrogenic preterm delivery), the models were not adjusted for preeclampsia. *Abbreviations*: *CI* confidence interval, *HR* hazard ratio, *SGA* small for gestational age

**Table 3:** Hazard ratios for maternal end-stage kidney disease by history of preterm delivery, among women delivering between 1973 and 2012 in Sweden (*n* = 1,943,716)

	End-stage kidney disease (*N* = 1268)		
	*n*	Minimally adjusted	Fully adjusted
		HR (95% CI)	HR (95% CI)
Exposure 1			
Term delivery (≥ 37 weeks)	1051	1.0	1.0
Preterm delivery (< 37 weeks)	217	2.56 (2.21–2.97)	2.22 (1.90–2.58)
Exposure 2			
Term delivery (≥ 37 weeks)	1051	1.0	1.0
Moderate preterm delivery (32–36 + 6 weeks)	174	2.36 (2.01–2.78)	2.08 (1.76–2.45)
Very preterm delivery (28–31 + 6 weeks)	30	3.81 (2.67–5.45)	2.90 (2.02–4.16)
Extremely preterm delivery (< 28 weeks)	13	4.15 (2.35–7.33)	3.61 (2.03–6.39)
Exposure 3			
Full term delivery (39–41 + 6 weeks)	584	1.0	1.0
Post-term delivery (≥ 42 weeks)	146	0.97 (0.81–1.16)	1.13 (0.94–1.36)
Early term delivery (37–38 + 6 weeks)	321	1.44 (1.26–1.66)	1.50 (1.31–1.73)
Moderate preterm delivery (32–36 + 6 weeks)	174	2.61 (2.20–3.10)	2.40 (2.02–2.86)
Very preterm delivery (28–31 + 6 weeks)	30	4.22 (2.94–6.05)	3.37 (2.34–4.87)
Extremely preterm delivery (< 28 weeks)	13	4.60 (2.60–8.16)	4.21 (2.37–7.49)
Exposure 4			
Term delivery (≥ 37 weeks)	1051	1.0	1.0
Spontaneous preterm delivery (< 37 weeks)	142	2.01 (1.68–2.39)	1.99 (1.67–2.38)
Iatrogenic preterm delivery—preeclampsia (< 37 weeks)	33	7.70 (5.41–10.97)	6.70 (4.70–9.56)
Iatrogenic preterm delivery—SGA (< 37 weeks)	25	3.84 (2.58–5.71)	3.72 (2.49–5.53)
Iatrogenic preterm delivery—preeclampsia and SGA (< 37 weeks)	17	6.72 (4.15–10.86)	6.25 (3.86–10.11)
Exposure 5			
Two term deliveries (≥ 37 weeks)	350	1.0	1.0
One preterm (< 37 weeks) and one term delivery (≥ 37 weeks)	75	2.71 (2.11–3.48)	2.12 (1.64–2.74)
Two preterm deliveries (< 37 weeks)	8	2.61 (1.29–5.26)	2.02 (1.00–4.08)

Hazard ratios represent separate Cox regression models for associations between preterm delivery and maternal end-stage kidney disease. Each exposure variable (1–5) represents different categories used to define gestational age at delivery. For all categories, preterm delivery was a time-dependent variable, where exposure status was based on the earliest gestation of any previous delivery. Minimally adjusted models controlled for year of delivery. Fully adjusted models controlled for year of delivery, maternal age, country of origin, education level, parity, inter-pregnancy interval, maternal BMI, smoking in pregnancy, exposure to gestational diabetes (time-dependent), and preeclampsia (time-dependent). In the analyses involving exposure 4 (spontaneous vs. iatrogenic preterm delivery), the models were not adjusted for preeclampsia. *Abbreviations*: *CI* confidence interval, *HR* hazard ratio, *SGA* small for gestational age

**Fig. 1:**
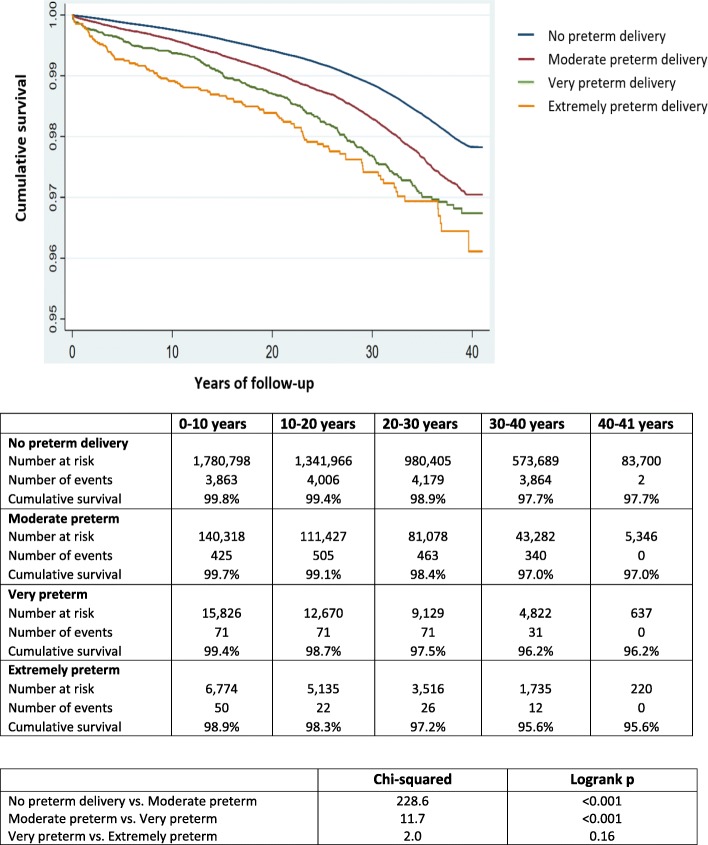
The Kaplan-Meier survival curves for risk of chronic kidney disease among women by exposure to moderate, very, or extremely preterm delivery between 1973 and 2012 in Sweden

Figure 1 shows the differences in the Kaplan-Meier curves for CKD across categories of gestational age (moderate/very/extremely preterm delivery). Relative to those who delivered at term (≥ 37 weeks), women who had at least one extremely preterm delivery (< 28 weeks) were at higher risk of CKD (aHR 1.84, 95% CI 1.52–2.22) and ESKD (aHR 3.61, 95%CI 2.03–6.39), respectively (exposure 2, Tables 2 and 3).

Women who experienced early term deliveries (37–38 + 6 weeks) were also at increased risk of CKD (aHR 1.19, 95% CI 1.15–1.24) and ESKD (aHR 1.50, 95% CI 1.31–1.73) compared to women who only had full term deliveries (39–41 + 6 weeks) (exposure 3, Tables 2 and 3).

### History of spontaneous or iatrogenic preterm delivery

**Fig. 2:**
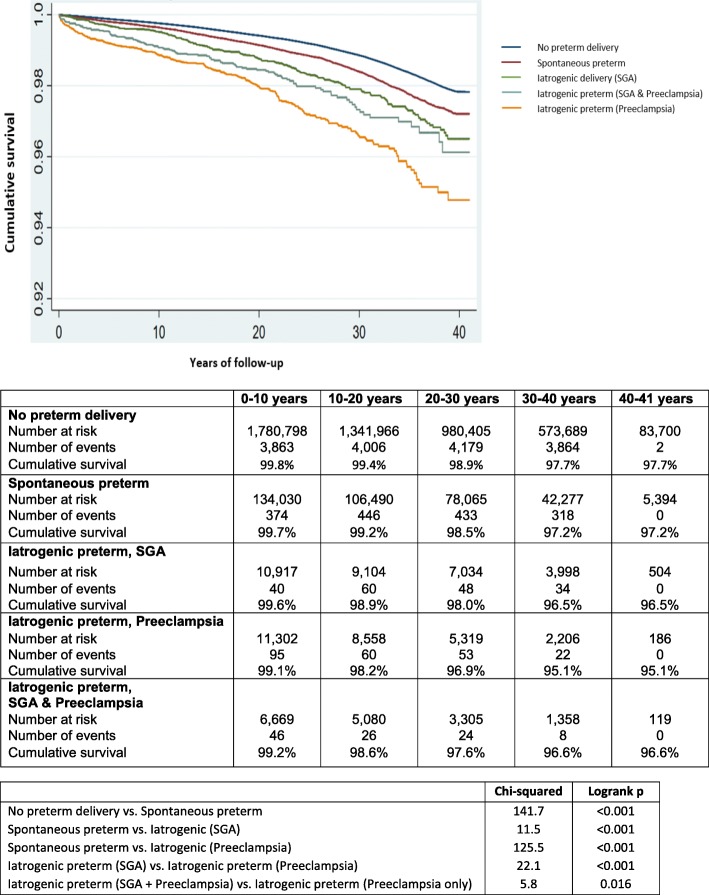
The Kaplan-Meier survival curves for risk of chronic kidney disease among women by exposure to spontaneous or iatrogenic preterm delivery between 1973 and 2012 in Sweden

Figure 2 shows the differences in the Kaplan-Meier survival curves for CKD across categories of spontaneous vs. iatrogenic preterm delivery. Compared to women who delivered at term (≥ 37 weeks), the risk of CKD and ESKD was highest in those who experienced preterm delivery (< 37 weeks) complicated by preeclampsia (for CKD, aHR 2.81, 95% CI 2.46–3.20; for ESKD, aHR 6.70, 95% CI 4.70–9.56) (exposure 4, Tables 2 and 3). Importantly, spontaneous preterm delivery (< 37 weeks) was also associated with increased risk of both CKD (aHR 1.32, 95% CI 1.25–1.39) and ESKD (aHR 1.99, 95% CI 1.67–2.38) independent of all other factors (exposure 4, Tables 2 and 3).

### Sensitivity analysis

All analyses were repeated for deliveries occurring between 1982 and 2012, when data became available on maternal BMI and smoking. No meaningful differences were observed between preterm delivery and CKD in these analyses (Table 4). Stronger associations were observed between extremely preterm delivery (< 28 weeks) and ESKD (aHR 5.14, 95% CI 2.81–9.40), and for preterm delivery + SGA and ESKD (aHR 4.89, 95% CI 3.33–8.40), but these associations were based on relatively few ESKD outcomes. The 95% CIs did not differ significantly from the corresponding 95% CIs in the main analysis.

**Table 4:** Hazard ratios for maternal chronic kidney disease and end-stage kidney disease by history of preterm delivery, restricted to women delivering between 1982 and 2012 in Sweden (*n* = 1,337,133)

	Chronic kidney disease (*N* = 10,922)		End-stage kidney disease (*N* = 547)	
	*n*	Fully adjusted	*n*	Fully adjusted
		HR (95% CI)		HR (95% CI)
Exposure 1				
Term delivery (≥ 37 weeks)	9428	1.0	424	1.0
Preterm delivery (< 37 weeks)	1494	1.39 (1.32–1.47)	123	2.28 (1.85–2.81)
Exposure 2				
Term delivery (≥ 37 weeks)	9428	1.0	424	1.0
Moderate preterm delivery (32–36 + 6 weeks)	1217	1.34 (1.26–1.42)	92	2.03 (1.61–2.56)
Very preterm delivery (28–31 + 6 weeks)	182	1.59 (1.37–1.84)	21	3.17 (2.01–5.01)
Extremely preterm delivery (< 28 weeks)	95	2.05 (1.67–2.51)	10	5.14 (2.81–9.40)
Exposure 3				
Full term delivery (39–41 + 6 weeks)	5032	1.0	213	1.0
Post-term delivery (≥ 42 weeks)	1202	1.04 (0.97–1.11)	48	1.01 (0.74–1.39)
Early term delivery (37–38 + 6 weeks)	3194	1.21 (1.15–1.26)	163	1.48 (1.20–1.82)
Moderate preterm delivery (32–36 + 6 weeks)	1217	1.44 (1.35–1.53)	92	2.36 (1.83–3.04)
Very preterm delivery (28–31 + 6 weeks)	182	1.71 (1.47–1.98)	21	3.70 (2.32–5.90)
Extremely preterm delivery (< 28 weeks)	95	2.20 (1.80–2.70)	10	5.98 (3.24–11.03)
Exposure 4				
Term delivery (≥ 37 weeks)	9428	1.0	424	1.0
Spontaneous preterm delivery (< 37 weeks)	1084	1.31 (1.23–1.40)	71	1.89 (1.46–2.44)
Iatrogenic preterm delivery—preeclampsia (< 37 weeks)	188	2.76 (2.39–3.19)	22	6.52 (4.19–10.14)
Iatrogenic preterm delivery—SGA (< 37 weeks)	134	1.79 (1.51–2.13)	19	4.89 (3.33–8.40)
Iatrogenic preterm delivery—preeclampsia and SGA (< 37 weeks)	88	2.18 (1.77–2.69)	11	5.91 (3.24–10.76)
Exposure 5				
Two term deliveries (≥ 37 weeks)	4208	1.0	189	1.0
One preterm (< 37 weeks) and one term delivery (≥ 37 weeks)	529	1.32 (1.21–1.45)	37	1.70 (1.18–2.44)
Two preterm deliveries (< 37 weeks)	72	1.43 (1.13–1.81)	7	2.66 (1.24–5.69)

Hazard ratios represent separate Cox regression models for associations between preterm delivery and maternal chronic kidney disease or end-stage kidney disease. Each exposure variable (1–5) represents different categories used to define gestational age at delivery. For all categories, preterm delivery was a time-dependent variable, where exposure status was based on the earliest gestation of any previous delivery. Minimally adjusted models controlled for year of delivery. Fully adjusted models controlled for year of delivery, maternal age, country of origin, education level, parity, inter-pregnancy interval, maternal BMI, smoking in pregnancy, exposure to gestational diabetes (time-dependent), and preeclampsia (time-dependent). In the analyses involving exposure 4 (spontaneous vs. iatrogenic preterm delivery), the models were not adjusted for preeclampsia. *Abbreviations*: *CI* confidence interval, *HR* hazard ratio, *SGA* small for gestational age

## Discussion

### Statement of principal findings

This study aimed to determine whether women who deliver preterm infants are at increased long-term risk of CKD and ESKD. The results suggest that preterm delivery may be considered as a marker of heightened risk of future CKD and ESKD in parous women, independently of causal mechanisms. This finding was consistent across various definitions of gestational age and was strongest in those with very/extremely preterm deliveries. Mothers exposed to early term delivery, at 37–38 + 6 weeks’ gestation, were also at increased risk of future renal disease relative to women who delivered at full term (39–41 + 6 weeks).

Women who experienced iatrogenic preterm delivery were at the highest risk of subsequent CKD or ESKD, and this may be largely driven by the effects of preeclampsia or SGA, or by the cumulative effect of preterm delivery in the context of these factors. However, our results suggest that women who had spontaneous preterm delivery were also at increased risk of CKD and ESKD irrespective of obstetric comorbidities or other factors. Given that the majority of preterm deliveries are spontaneous [14], it is relevant to consider whether these women should be informed of their heightened relative risk, and whether their history of preterm deliveries should be considered as part of their overall risk profile for chronic disease.

### Interpretation

There is a small body of literature which has examined independent associations between preterm delivery and maternal renal disease [21]. Longitudinal studies from Canada, Israel, and Norway have each reported that preterm delivery increases the risk of maternal ESKD, but they have been limited by incomplete adjustment for confounders [11–13]. Other cohort studies have been restricted to women with pre-existing diabetes [9] or renal disease [10], and cannot be generalised to wider populations. Our study builds on existing research by controlling for a broader range of covariates, including maternal smoking, BMI, and inter-pregnancy interval, and by excluding women with pre-existing medical comorbidities, which may have otherwise increased the risk of CKD. Moreover, previous studies focused on ESKD [9–11, 13] or kidney-related hospitalisation [12] as their outcome variable. To our knowledge, the current study is the first to report the association between preterm delivery and maternal CKD.

Only one previous study on this topic stratified preterm deliveries according to whether they were spontaneous or iatrogenic [12]. Although spontaneous preterm delivery was independently associated with CKD and ESKD in our study, the results suggest that preeclampsia, and to a lesser extent SGA, has a greater impact on renal risk than preterm delivery. Preeclampsia has been reported to increase the risk of maternal CKD [22–25] and ESKD [11, 13, 15, 22] previously.

The mechanisms underlying the independent associations between spontaneous preterm delivery and maternal CKD/ESKD are uncertain. Intuitively, it seems plausible that this is a manifestation of subclinical predisposition to renal and cardiometabolic disease. Women with a history of preterm delivery are at higher risk of developing hypertension, diabetes, and hypercholesterolaemia [26], as well as CVD [4–6], and these factors may all increase the subsequent risk of CKD and ESKD. It is also possible that inflammatory processes associated with spontaneous preterm delivery increase the risk of endothelial dysfunction and subclinical vascular disease, which in turn increase the risk of subsequent CKD and ESKD [27, 28]. Women with spontaneous preterm deliveries are considered to have a pro-inflammatory phenotype [29]. They may have higher C-reactive protein (CRP) levels in pregnancy [28, 30], and CRP is a strong predictor of later CKD risk [31]. Thus, inflammatory factors may underlie a woman’s predisposition to deliver at earlier gestation and her later susceptibility to renal disease.

### Strengths and weaknesses of the study

This study has several strengths. The use of a large national cohort of pregnant women with a 41-year follow-up period reduced the possibility of selection bias and provided statistical power to examine the effect of gestational age independently of a wide range of recognised risk factors for maternal renal disease. The size of our cohort allowed us to provide robust effect estimates for spontaneous and iatrogenic preterm delivery. Our data were retrieved from national registers with mandatory reporting, thereby excluding the possibility of recall bias. We used time-dependent covariates which allowed for changes in exposure status over time, and are more representative of women’s cumulative exposure to obstetric risks during their reproductive lifetime.

Gestational age was identified from the MBR based on maternal report of LMP in the 1970s and early 1980s, and using ultrasound estimation from 1982 onwards where available. LMP is a less accurate method of predicting a woman’s delivery date than ultrasonography [32]. It is possible that some term births may have been misclassified and included in earlier categories of preterm delivery. This may have led to underestimation of the observed risks, but is unlikely to have affected the overall results. When ultrasound dating is used, first trimester crown-rump length is regarded as the best parameter for determining gestational age, and should be used where possible [33–36]. In our study, the standard ultrasound method to date pregnancies was through combination of biparietal diameter and femoral length in the second trimester. Although this method has been validated previously [37], we cannot exclude the possibility of misclassification of some term or preterm births. Pregnancy dating guidelines in Sweden have been updated in recent years and recommend the use of first trimester ultrasound where possible [34], but evidence suggests ongoing variation in adherence to these guidelines [38].

Our study population was primarily Swedish/Caucasian, and this may limit generalisability of the findings. Black women have higher prevalence of preterm delivery than Caucasian women [39], and the causes of preterm delivery may vary by ethnicity or change over time.

The majority of cases of CKD and ESKD were identified using ICD-coded diagnoses in the NPR. There was no specific ICD-8 code for ESKD before 1986, and although some individuals may have been captured as CKD in the NPR, ESKD cases may have been underestimated as a result. Data collection was also less comprehensive in the NPR before national coverage was achieved in 1987, and outpatient diagnoses were only recorded from 2001 onwards [40]. The SRR only collected ESKD data from 1991 onwards, and CKD data from 2007 onwards; thus, we cannot exclude the possibility of immortal time bias in our study.

The SRR has almost complete coverage of ESKD cases in Sweden, but coverage of CKD cases is lower [41]. There was a relatively low prevalence of CKD among women in our study, possibly due to a combination of under-ascertainment of cases from the SRR and NPR, strict exclusion criteria, and the relative youth of the cohort involved. It is likely that a considerable number of women with renal disease were never diagnosed or ascertained by either the NPR or the SRR. However, an external review has reported high positive predictive values for most diagnoses recorded in the NPR, despite lower sensitivity levels [40]. Thus, it is likely that those who were diagnosed with CKD or ESKD in the dataset were valid diagnoses.

Most variables in the dataset had almost complete data, but there were large amounts of missing data on maternal BMI and smoking. Missing data can significantly affect prevalence estimates, but only have a slight impact on risk estimates assuming the lack of information is random [42]. In our dataset, the availability of data on maternal BMI and smoking varied by year of delivery, because these covariates were only collected from 1982 onwards. We used missing indicator variables, and adjusted for year of delivery in all models, to control for this. We also conducted sensitivity analyses restricted to pregnancies after 1982, and most results were not substantially different.

There was no suitable ICD-8 code available for gestational diabetes in the NPR. We could not ascertain those who may have been diagnosed before 1986, although this proportion is likely to be small. Finally, we excluded women on the basis of pre-existing comorbidities at baseline, including chronic hypertension. This information came from both the birth and hospital registers, but the NPR has low sensitivity for chronic hypertension. Thus, we cannot exclude the possibility of unmeasured confounding.

### Implications

It is likely that the absolute risk of ESKD remains very low in women with a history of preterm delivery (with or without preeclampsia), despite the high relative risks observed in this study, since ESKD is a rare outcome. By contrast, modest increases in the risk of CKD in women exposed to spontaneous preterm deliveries or early term births may be important from a population perspective. Early term births (at 37–38 + 6 weeks) account for about 22% of all births in high-income countries [43], and the majority of preterm and early term births occur spontaneously [14, 44].

Obstetric history is easy to collect in the clinical setting, and it could be of potential use for renal risk stratification in women. Existing prediction models for CKD and ESKD have not taken obstetric factors into consideration [45]. Further research is warranted to elucidate whether incorporating history of spontaneous preterm delivery, and other relevant obstetric factors, adds incremental value or clinically relevant information to the overall risk of renal disease for women when other demographic and cardiometabolic risk factors have been accounted for.

## Conclusions

Women with a history of preterm delivery are at increased risk of maternal CKD and ESKD. The risk of renal disease is highest among women who have experienced very or extremely preterm deliveries, and those whose preterm delivery is medically indicated. Women who experience spontaneous preterm delivery are at increased risk of renal disease independently of preeclampsia and SGA. Preterm delivery may act as an important risk marker of adverse renal outcomes in the years and decades following pregnancy irrespective of causal mechanisms.

## Supplementary information

**Additional file 1: Supplementary Figure S1.** Timeline of study design.

**Additional file 2: Supplementary Table S1.** ICD codes used for disease definitions.

**Additional file 3: Supplementary Figure S2.** Flow chart illustrating construction of study cohort.

## Data Availability

Data are from the Swedish Medical Birth Register, National Patient Register, and Swedish Renal Register. Data cannot be put into a public data repository due to Swedish confidentiality regulations for registry data. Details on the application procedures for data usage are available on the homepages of the respective registries: the Medical Birth Register (https://www.socialstyrelsen.se/en/statistics-and-data/registers/alla-register/the-swedish-medical-birth-register/), the National Patient Register (https://www.socialstyrelsen.se/en/statistics-and-data/registers/alla-register/the-national-patient-register/), and the Swedish Renal Register (https://www.medscinet.net/snr/).

## Abbreviations

aHR: Adjusted hazard ratio
BMI: Body mass index
CI: Confidence interval
CKD: Chronic kidney disease
CRP: C-reactive protein
CVD: Cardiovascular disease
ESRD: End-stage renal disease
ICD: International Classification of Diseases
IUGR: Intrauterine growth restriction
LMP: Last menstrual period
MBR: Medical Birth Register
NPR: National Patient Register
SGA: Small for gestational age
SRR: Swedish Renal Register
