# Supplementary figures and images for: Risk of long-term renal disease in women with a history of preterm delivery: a population-based cohort study
Source: BMC Med. 2020 Apr 1;18:66. doi: 10.1186/s12916-020-01534-9 (PMC7110747; doi:10.1186/s12916-020-01534-9)

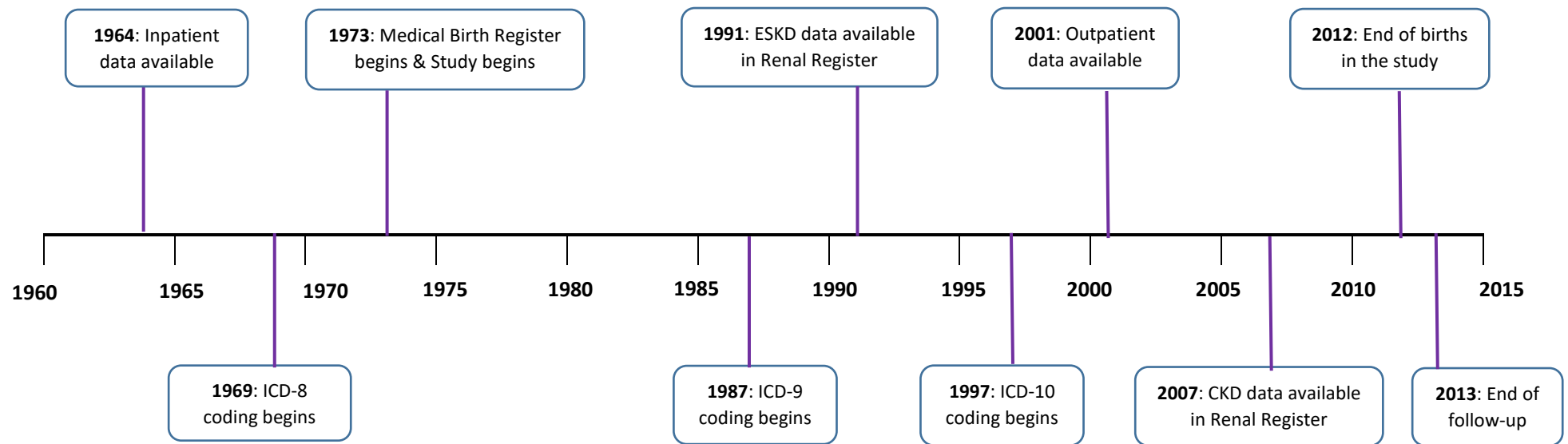

**Supplementary Figure S1. Timeline of study design**

Supplement: Supplementary file 1 — Additional file 1: Supplementary Figure S1. Timeline of study design. [file 12916_2020_1534_MOESM1_ESM.pdf]

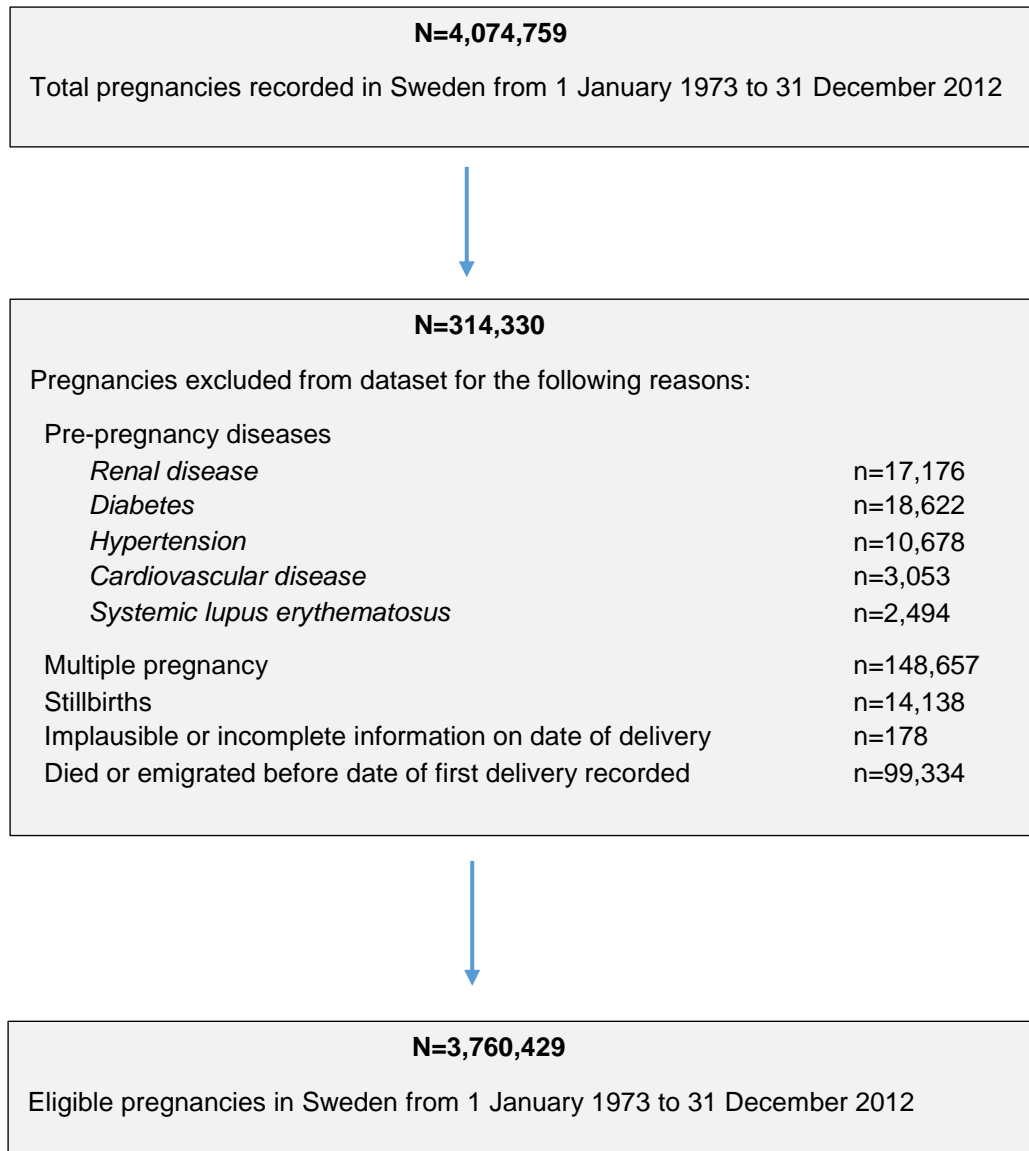

**Supplementary Figure S2. Flow chart illustrating construction of study cohort**

Supplement: Supplementary file 3 — Additional file 3: Supplementary Figure S2. Flow chart illustrating construction of study cohort. [file 12916_2020_1534_MOESM3_ESM.pdf]
