# Supplementary material for: Risk of long-term renal disease in women with a history of preterm delivery: a population-based cohort study
Source: BMC Med. 2020 Apr 1;18:66. doi: 10.1186/s12916-020-01534-9 (PMC7110747; doi:10.1186/s12916-020-01534-9)
Supplement: Supplementary file 2 — Additional file 2: Supplementary Table S1. ICD codes used for disease definitions. [file 12916_2020_1534_MOESM2_ESM.docx]

**Supplementary Table S1. ICD codes used for disease definitions**

|  | **ICD-8 codes**  **(1973-1986)** | **ICD-9 codes**  **(1987-1996)** | **ICD-10 codes**  **(1997-2013)** |
| --- | --- | --- | --- |
| Any pre-existing chronic/end-stage kidney disease, congenital or genetic causes of renal disease (for exclusion) | 403-404, 580-589, 753 | 403-404, 580-589, 753, V42A, V45B, V56A, V56W | N00-N08, N10-N19, P960, Q271, Q272, Q60-Q63, Q878, Z49, Z992, Z940, T861 |
| Chronic kidney disease (outcome) | 403-404, 581-583, 585-588 | 403-404, 581-583, 585-588, V42A, V45B, V56A, V56W | N01-N06, N08, N11-N13, N15-16, N18-19, Z49, Z992, Z940, T861 |
| End-stage kidney disease (outcome) | None | V42A, V45B, V56A, V56W | N185, Z49, Z992, Z940, T861 |
| Cardiovascular disease | 393-398  410-436 | 393-398  410-436 | I16-I64  G45 |
| Hypertension | 401-405 | 401-405 | I10-I15 |
| Diabetes (type 1 or type 2) | 250 | 250 | E10-E14 |
| Systemic lupus erythematosus | 73410 | 710A | M32 |
| Preeclampsia | 63700, 63703, 63704, 63709, 63710, 63719, 63790, 63799 | 642E, 642F, 642G | O140, O141, O141A,  O141B, O141C, O141X, O142, O149, O150, O151, O152, O159 |
| Gestational diabetes | None | 648W | O244 |
